# Pediatric Anaphylaxis in the Emergency Department: From Mechanisms to Personalized Care

**DOI:** 10.7759/cureus.94988

**Published:** 2025-10-20

**Authors:** Ghaith K Mansour, Yara AlGoraini

**Affiliations:** 1 Pharmaceutical Sciences, Alfaisal University College of Pharmacy, Riyadh, SAU; 2 Pediatric Emergency, King Fahad Medical City, Riyadh, SAU

**Keywords:** emergency medicine, epinephrine pharmacokinetics, mast cell degranulation, pediatric anaphylaxis, precision dosing

## Abstract

Pediatric anaphylaxis remains a time-critical emergency in children due to its unpredictable onset and risk of rapid deterioration. The reaction involves mast cell activation through both IgE-dependent and non-IgE pathways, leading to widespread immune responses. Effective management relies on the timely administration of epinephrine, though age-related factors complicate dosing and delivery. Emerging approaches such as alternative administration routes and individualized treatment strategies may improve outcomes. Precision-based care and multidisciplinary coordination are essential to reduce morbidity and mortality in this vulnerable population.

## Introduction and background

Pediatric anaphylaxis is one of the most time-critical emergencies in pediatric emergency medicine, affecting 1-3% of the global pediatric population, with incidence steadily increasing [1-3]. It is a severe, systemic hypersensitivity reaction that can progress to cardiovascular collapse and death within minutes if untreated [2]. Across multiple regions, pediatric anaphylaxis has climbed sharply, often reported as a five- to seven-fold rise in hospital admissions, with food allergens the dominant trigger; rates are higher in boys, vary by region (e.g., U.S. Northeast), and are influenced by sociodemographic and hospital-setting factors [3]. The pathophysiology is complex and differs significantly from adults, requiring age-specific therapeutic approaches [4]. Advances in mast cell biology, epinephrine pharmacokinetics, and immunological determinants of severity now have direct implications for clinical practice, particularly for dosing strategies, alternative delivery methods, and precision medicine approaches [1].

## Review

Pathophysiology

Pediatric anaphylaxis represents a rapidly evolving systemic hypersensitivity reaction that can escalate to cardiovascular collapse if not promptly recognized and treated. The underlying mechanism centers on mast cell and basophil activation, occurring through both IgE-dependent and non-IgE-mediated pathways. In the classical IgE-mediated pathway, allergens crosslink IgE antibodies bound to FcεRI receptors on mast cells, triggering calcium influx and mediator release [5,6]. These mediators, namely, histamine, tryptase, leukotrienes, and prostaglandins, cause vasodilation, bronchoconstriction, mucosal edema, and gastrointestinal hypermotility. In contrast, non-IgE-mediated activation may result from direct pharmacologic triggers such as opioids, vancomycin, and radiocontrast media, or through complement activation and pseudoallergic mechanisms [7]. Recent studies highlight mitochondrial dysfunction and oxidative stress as amplifiers of mast cell activation, where loss of mitochondrial membrane potential and excessive reactive oxygen species (ROS) generation exacerbate systemic mediator release [1]. Experimental data link cardiomyocyte mitochondrial impairment to left ventricular dysfunction and lipid peroxidation during anaphylactic shock, suggesting that pediatric anaphylaxis is not merely an allergic reaction but a systemic metabolic and inflammatory crisis [1,7-9].

Molecular Mechanisms of Mast Cell Activation

The pathophysiology of pediatric anaphylaxis is driven by rapid mast cell and basophil degranulation through both IgE-dependent and independent pathways [5]. In the classical IgE-mediated mechanism, allergen-IgE complexes crosslink high-affinity FcεRI receptors on mast cells, triggering intracellular calcium mobilization and subsequent degranulation [6]. Recent evidence shows that this process depends on intact mitochondrial respiratory function and membrane potential, with ROS acting as critical mediators. Mitochondrial dysfunction and oxidative stress, therefore, play pivotal roles in anaphylactic mast cell activation. Experimental studies of cardiomyocyte metabolism during anaphylactic shock further demonstrate cardiac dysfunction linked to left ventricular mitochondrial impairment and lipid peroxidation [1], suggesting that anaphylaxis involves systemic mitochondrial dysfunction beyond localized mast cell activation.

Novel Regulatory Pathways

Recent studies highlight galectin-9 (Gal-9) as a key regulator of mast cell degranulation, with endogenous sulfur dioxide (SO₂) acting as a natural inhibitor by sulfenylating Gal-9 at cysteine 74. This modification has emerged as a potential therapeutic target, as SO₂ supplementation reverses excessive degranulation in experimental models [7]. In addition, short-chain fatty acids such as butyrate and valerate have been shown to suppress IgE-mediated mast cell activation via GPR109A signaling and enhanced prostaglandin E₂ (PGE₂) release [8].

Compound Exocytosis and Mediator Release

During anaphylaxis, mast cells predominantly secrete through compound exocytosis, a specialized process in which secretory granules fuse before releasing their contents [5]. This allows the rapid discharge of preformed mediators such as histamine, tryptase, heparin, and cytokines that drive the systemic manifestations of the reaction. Notably, mast cell-derived heparin provides negatively charged surfaces that facilitate factor XII binding and auto-activation, thereby linking degranulation to activation of the contact and coagulation pathways [9].

Pharmacotherapy and drug treatments

The cornerstone of management in pediatric anaphylaxis remains epinephrine, the only medication proven to reduce mortality [3,10]. Intramuscular injection into the anterolateral thigh at a dose of 0.01 mg/kg concentration 1:1,000 (maximum 0.5 mg) is universally recommended. Epinephrine’s mechanism combines α1-adrenergic vasoconstriction, β2-mediated bronchodilation, and mast cell stabilization, counteracting the core pathophysiologic processes of anaphylaxis. Despite clear evidence, underuse and delayed administration continue to contribute to preventable morbidity and mortality. Infants under 15 kg present a dosing challenge, as standard 0.15 mg autoinjectors may deliver supratherapeutic doses; however, allergy societies support their use when smaller-dose devices are unavailable, as withholding epinephrine poses a greater risk [11]. Updated guidelines (2023-2024) now recommend 0.1 mg autoinjectors for 7.5-14 kg, 0.15 mg for 15-29 kg, 0.3 mg for 30-49 kg, and 0.5 mg for ≥50 kg to achieve age-appropriate plasma levels. About 30-40% of children require more than one dose, emphasizing the need for access to multiple autoinjectors [12]. In refractory anaphylaxis, where symptoms persist after two intramuscular doses, intravenous infusion is indicated using 1 mg epinephrine diluted in 1,000 mL (1 µg/mL) of saline, initiated at 0.1 µg/kg/minute and titrated to effect, under continuous cardiac monitoring [13]. New needle-free formulations, such as sublingual tablets and intranasal sprays, are emerging as effective, faster-acting, and more acceptable alternatives for children [13,14].

Epinephrine: First-Line Therapy

Intramuscular epinephrine remains the first-line treatment for pediatric anaphylaxis, administered at a 0.01 mg/kg concentration 1:1,000 (maximum 0.5 mg) into the anterolateral thigh. Updated international recommendations (2023-2024) have refined autoinjector dosing to better match pediatric weight ranges and pharmacokinetic data. A 0.1 mg autoinjector is now preferred for children weighing 7.5-14 kg, minimizing the risk of overdosing seen with 0.15 mg devices. For children 15-29 kg, the 0.15 mg autoinjector remains standard, while those 30-49 kg should receive 0.3 mg, and patients ≥50 kg should use 0.5 mg autoinjectors, as higher doses achieve superior plasma concentrations and hemodynamic responses. Infants <7.5 kg should continue to receive 0.01 mg/kg concentration 1:1,000 intramuscularly via syringe until suitable autoinjectors become available. These updates, supported by Kim et al. (2023), replace the earlier 2015 Canadian Society of Allergy and Clinical Immunology guidance and emphasize precision-based, weight-adjusted epinephrine delivery for optimal safety and efficacy in children [11]. About 30-40% of children require more than one epinephrine dose, underscoring the need for multiple autoinjectors [12]. Refractory anaphylaxis, occurring in roughly 1% of severe cases despite two intramuscular doses, warrants intravenous infusion using a 1:10,000 (0.1 mg/mL) dilution under critical care supervision [3].

Alternative Delivery Systems

Novel epinephrine delivery systems offer solutions to pediatric-specific challenges, particularly needle phobia, which contributes to the underuse of autoinjectors. Sublingual rapidly disintegrating tablets containing 30 mg of epinephrine have shown bioequivalent peak plasma concentrations (16.7 ± 1.9 ng/mL) compared to intramuscular 0.15 mg doses (18.8 ± 1.9 ng/mL) in preclinical studies, with therapeutic levels reached faster (≈21 minutes vs. 36 minutes) and improved acceptability in children [13]. Intranasal formulations are also under investigation, with pharmacokinetic studies demonstrating adequate systemic absorption in pediatric patients with allergic reaction histories [14]. Together, these needle-free alternatives may enhance treatment compliance and improve outcomes in children where injection-related barriers delay timely intervention.

Adjunctive Therapies

Adjunctive treatments for pediatric anaphylaxis include H1 and H2 antihistamines, corticosteroids, and bronchodilators, but these should never delay or replace epinephrine administration [2]. Diphenhydramine (H1 antagonist) is given at 1 mg/kg/dose intramuscularly or intravenously (maximum 50 mg per dose), while cetirizine may be used orally at 0.25 mg/kg (maximum 10 mg). For H2 blockade, ranitidine, though less commonly used now, was traditionally dosed at 1 mg/kg intravenously (IV) (maximum 50 mg), and famotidine may be given at 0.5 mg/kg IV or PO (maximum 20 mg). Corticosteroids such as methylprednisolone are administered at 1-2 mg/kg IV every six hours (maximum 125 mg), and prednisolone or prednisone at 1 mg/kg orally (maximum 60 mg) may be used for follow-up to reduce the risk of biphasic reactions. Bronchodilators such as salbutamol (albuterol) are useful for persistent bronchospasm, delivered by nebulization at 2.5 mg (<20 kg) or 5 mg (≥20 kg) every 20 minutes as needed. Corticosteroids may help reduce the risk of biphasic reactions, which occur in 5-20% of cases, usually within four to six hours of initial symptom resolution. Therefore, current guidelines recommend observation for at least four to six hours following epinephrine administration, although the optimal duration remains debated [15].

Clinical immunology

The clinical presentation and immune response in anaphylaxis vary considerably by age, reflecting developmental differences in mast cell distribution, immune system maturation, and tissue reactivity. Infants and toddlers tend to show gastrointestinal manifestations such as vomiting and abdominal pain, whereas older children exhibit respiratory compromise and cardiovascular instability [4,16]. Understanding these age-specific immunological profiles allows clinicians to anticipate organ involvement and tailor interventions accordingly. Diagnostic advances include the use of serum tryptase as a post-event biomarker, though it may remain normal in food-related cases or those treated early with epinephrine [17]. Emerging biomarkers, such as mast cell protease-1 (MCPT-1) and cytokine panels, are being investigated for their ability to enhance diagnostic precision and severity prediction [18,19]. Moreover, immunomodulatory strategies such as cannabidiol (CBD) have shown inhibition of IgE-mediated mast cell degranulation via FcεRI pathway modulation [6], while probiotic interventions with *Lactiplantibacillus plantarum* strains can reduce IgE synthesis and mast cell activity in experimental models [19]. Together, these findings point toward an evolving paradigm that integrates immunological profiling and targeted modulation in pediatric anaphylaxis care.

Age-Related Immunological Differences

Pediatric anaphylaxis exhibits age-specific patterns reflecting developmental immune differences. Infants and toddlers are more likely to present with gastrointestinal symptoms, while school-aged children more often show respiratory involvement. These variations align with differences in mast cell distribution, tissue responsiveness, and immune system maturation [4,16].

Biomarker Development

Recent advances have identified novel biomarkers for diagnosis and severity assessment in anaphylaxis. Serum tryptase remains the most established marker, although elevated levels may persist for several hours post-reaction [18]. Emerging biomarkers, including MCPT-1 and various cytokine signatures, may enable earlier recognition and improved severity stratification [17-19].

Immunomodulatory Interventions

Experimental therapies targeting mast cell function show promise for prevention and treatment. CBD significantly inhibits IgE-mediated mast cell degranulation by suppressing FcεRI downstream signaling and calcium mobilization, independent of cannabinoid receptors [6]. Probiotic interventions, particularly *Lactiplantibacillus plantarum* strains, attenuate mast cell degranulation and reduce total IgE levels in experimental models [19].

Component-Resolved Diagnostics

Component-resolved diagnostics provide more precise allergen identification and risk stratification, supporting personalized management strategies. These tools may also help predict severity based on reactivity to specific allergen components, and their integration into emergency care could improve both acute management and long-term prevention [20].

Clinical microbiology

Drug-induced anaphylaxis represents a significant proportion of pediatric emergency presentations, with β-lactam antibiotics and vancomycin among the most frequent culprits [16,21]. Vancomycin, while a cornerstone in treating resistant Gram-positive infections, is increasingly recognized as a cause of both IgE-mediated anaphylaxis and delayed hypersensitivity reactions. Notably, drug reaction with eosinophilia and systemic symptoms (DRESS) syndrome, systematically reviewed in recent literature, represents a severe and potentially fatal form of vancomycin-induced immune dysregulation, with mortality rates approaching 10% [22,23]. The immunopathogenesis involves drug metabolite binding to host proteins, forming hapten-carrier complexes that elicit IgE-mediated responses. Vancomycin can also trigger delayed hypersensitivity syndromes such as DRESS, characterized by rash, lymphadenopathy, and organ dysfunction, with mortality approaching 10%. This dual potential for immediate and delayed reactions underscores the importance of careful antibiotic selection and allergy documentation. The clinical challenge is further compounded by antimicrobial resistance, as avoidance of first-line β-lactams often necessitates broader-spectrum alternatives that promote resistance [17]. Beyond pathogen exposure, the gut microbiome exerts regulatory influence through short-chain fatty acids (e.g., butyrate, valerate) that suppress mast cell activation and enhance tolerance [8,19]. Moreover, rapid diagnostic assays in the emergency department help differentiate infectious mimics of anaphylaxis, reducing inappropriate antibiotic use and aligning with antimicrobial stewardship priorities.

Antimicrobial Considerations in Anaphylaxis

Drug-induced anaphylaxis represents a significant proportion of pediatric emergency presentations, with antibiotics being the most common culprits. β-lactam antibiotics, especially penicillins and cephalosporins, account for most cases and trigger IgE-mediated hypersensitivity via hapten-protein conjugates formed when antibiotic metabolites bind to carrier proteins [16,21].

Antimicrobial Resistance Implications

Managing pediatric patients with anaphylaxis to first-line antibiotics is particularly challenging in the era of rising antimicrobial resistance [17]. In cases such as suspected sepsis, clinicians must balance the urgent need for antimicrobial therapy against the risk of drug-induced anaphylaxis, often resorting to broader-spectrum agents that increase resistance pressure. Effective stewardship requires accurate allergy documentation and thoughtful alternative agent selection to minimize unnecessary broad-spectrum use and preserve antibiotic effectiveness [17].

Microbiome Interactions

The gut microbiome appears to influence susceptibility and severity of anaphylaxis by shaping immune development and mast cell function [19]. Short-chain fatty acids derived from commensal bacteria, including butyrate, exert protective effects by suppressing mast cell activation [8]. These findings raise the possibility of microbiome-targeted interventions to reduce anaphylaxis risk, although clinical applications remain investigational.

Diagnostic Microbiology in Emergency Settings

Rapid diagnostic testing plays a vital role in distinguishing anaphylaxis from sepsis and other systemic inflammatory conditions [17]. Point-of-care assays for bacterial and viral pathogens allow more precise antibiotic prescribing, reduce unnecessary antimicrobial exposure in patients with anaphylaxis mimics, and support stewardship efforts. Incorporating these rapid platforms into pediatric emergency workflows enhances timely care while maintaining stewardship priorities.

Pharmacokinetics and pharmacodynamics

Epinephrine pharmacokinetics in children differ substantially from adults because of age-dependent changes in absorption, distribution, metabolism, and clearance. Bioavailability in neonates is about 49% of adult levels, gradually increasing to full adult equivalence by approximately two years of age [23]. This maturation-dependent variability makes fixed-dose autoinjectors suboptimal for precise pediatric dosing. Recent advances in population pharmacokinetic and physiologically based pharmacokinetic modeling have improved dose prediction by integrating factors such as weight, age, and organ function [22,24,25]. These models underpin model-informed precision dosing (MIPD) strategies, which tailor therapy to individual pharmacologic and physiologic profiles [22]. Drug interactions also influence outcomes; β-blockers can blunt epinephrine’s therapeutic effect, while antihypertensive agents may modify hemodynamic responses [11,22,26,27]. Consequently, optimizing epinephrine dosing in pediatric anaphylaxis requires a precision-based framework rather than empirical fixed-dose regimens.

Age-Related Pharmacokinetic Variations

Pediatric pharmacokinetics differ substantially from adults due to developmental changes in absorption, distribution, metabolism, and elimination [22]. For epinephrine, bioavailability in infants is about 49% of adult levels at birth and reaches adult values by two years of age [23], underscoring the need for age-specific dosing adjustments to ensure therapeutic exposures.

Population Pharmacokinetic Modeling

Population pharmacokinetic and physiologically based pharmacokinetic models now inform pediatric dosing by integrating adult data with age-appropriate physiology [24,25]. These approaches support evidence-based guidelines where pediatric trial data are limited, helping refine emergency dosing recommendations.

Precision Dosing Strategies

MIPD incorporates weight, age, comorbidities, and concomitant medications to individualize therapy [11,26]. Combining therapeutic drug monitoring with real-time pharmacokinetic models may optimize outcomes while reducing adverse effects in critically ill children.

Bioavailability and formulation considerations

The bioavailability of intramuscular epinephrine varies with injection site, needle length, and adipose tissue thickness, emphasizing the need for pediatric-appropriate autoinjector design [4]. Sublingual and intranasal formulations provide more consistent absorption and better acceptability in children [13,14].

Drug Interactions and Comorbidity Effects

Children with cardiovascular comorbidities or those taking β-blockers may show reduced responsiveness to epinephrine, while some data suggest antihypertensive use may paradoxically lower dosing requirements [27]. These pharmacodynamic interactions must be carefully considered in emergency management.

Pharmacokinetics/Pharmacodynamics of epinephrine in pediatric anaphylaxis

Pharmacokinetic and pharmacodynamic data are central to refining epinephrine dosing accuracy in pediatric populations. After an intramuscular injection of 0.15 mg via an autoinjector, children typically reach peak plasma concentrations (Cmax) of 18.8 ± 1.9 ng/mL at around 36 minutes, which correlates with clinical improvement once plasma levels exceed 10 ng/mL [13,28,29]. However, pharmacokinetic behavior becomes nonlinear during stress responses, emphasizing the need for individualized dosing. Sublingual 30 mg epinephrine tablets have demonstrated comparable plasma levels achieved faster (~21 minutes), while intranasal formulations provide similarly rapid absorption [13,14]. Population pharmacokinetic/pharmacodynamic modeling predicts that children aged two to four years require roughly 40% of adult exposure for therapeutic levels, while those aged five to nine years require ~70% [28]. For older children nearing adult weight (>40 kg), higher doses (0.3-0.5 mg) are often necessary to reach optimal therapeutic thresholds [29]. In cases of refractory anaphylaxis, intravenous infusion at 0.1-1 µg/kg/minute using a 1 µg/mL solution (1 mg in 1,000 mL saline) is recommended under intensive monitoring [20]. Collectively, these insights underscore the transition from traditional one-size-fits-all dosing to precision pharmacology, integrating pharmacokinetic/pharmacodynamic modeling, clinical data, and emerging delivery systems to optimize outcomes in pediatric anaphylaxis.

Pharmacokinetic Parameters

After intramuscular administration of 0.15 mg epinephrine via an autoinjector, children reach peak plasma concentrations (Cmax) of 18.8 ± 1.9 ng/mL at ~36 minutes [13]. Distribution and clearance scale allometrically with body weight, but repeated dosing introduces nonlinear kinetics due to stress-induced physiological changes [28].

Pharmacodynamic Relationships

Epinephrine acts through α1-, α2-, β1-, and β2-adrenergic receptors, with cardiovascular effects such as increased heart rate, blood pressure, and cardiac output typically achieved at plasma concentrations above 10 ng/mL [3,29]. Age-related receptor sensitivity and distribution contribute to variability across pediatric groups.

Dose-Response Optimization

Recent trials in adolescents demonstrate superior plasma levels and hemodynamic responses with 500 μg versus 300 μg autoinjectors, suggesting current pediatric dosing may be inadequate for older children approaching adult weight [29]. Weight-based dosing algorithms guided by PK modeling may provide more precise targeting.

In cases of refractory anaphylaxis requiring intravenous epinephrine, a diluted infusion of 1 mg in 1,000 mL (1 µg/mL) using 0.9% saline is recommended. The infusion rate should start at 0.1 µg/kg/minute and be titrated every few minutes to a typical range of 0.1-1 µg/kg/minute according to clinical response and blood pressure. Continuous cardiac monitoring is mandatory, and the infusion should be prepared using a 1:10,000 (0.1 mg/mL) solution drawn from standard epinephrine ampoules for precise dosing in older children approaching adult weight. These protocols are supported by recent pediatric pharmacology and resuscitation guidelines emphasizing cautious titration to effect [11].

Alternative Route Pharmacokinetics

Sublingual 30 mg epinephrine achieves equivalent peak levels more rapidly (~21 minutes) compared with intramuscular delivery (~36 minutes), while intranasal formulations show similarly fast and consistent systemic absorption [13,14].

Population Pharmacokinetic-Pharmacodynamic Modeling

Advanced pharmacokinetic/pharmacodynamic models predict that children aged two to four years require ~40% of adult doses to achieve therapeutic exposures, while those aged five to nine years require ~70% [28]. These models support personalized dosing strategies that balance efficacy with safety.

Pathophysiology and molecular insights

Anaphylaxis in children is a rapidly evolving systemic reaction initiated by mast cell and basophil activation. While classical IgE-mediated crosslinking of FcεRI receptors remains the dominant pathway, increasing evidence supports the contribution of non-IgE mechanisms, mitochondrial dysfunction, and oxidative stress in amplifying the response. These processes promote calcium flux, ROS generation, and mediator release, linking mitochondrial health to the severity of the reaction. Novel regulators such as Gal-9 modification and microbiome-derived metabolites (e.g., short-chain fatty acids) are emerging as important modulators of mast cell stability. Collectively, these findings highlight that pediatric anaphylaxis involves systemic metabolic and immunological alterations, not solely localized allergic activation [1,7,8,19].

Clinical management and pharmacotherapy

Epinephrine administered intramuscularly remains the standard of care and is the only intervention proven to reduce mortality. Yet, challenges persist in pediatric populations, particularly in infants under 15 kg, where current autoinjector doses may be disproportionate to body weight. Multiple doses are often required, and refractory anaphylaxis, though uncommon, necessitates intravenous infusion under intensive monitoring. Adjunctive therapies, such as antihistamines, corticosteroids, and bronchodilators, may provide symptomatic relief but should never delay epinephrine. Observation protocols vary, but a minimum of four to six hours is generally recommended due to the risk of biphasic reactions. These considerations emphasize the importance of tailored emergency department pathways for children [29].

Diagnostic advances

Improved diagnostics are reshaping the approach to pediatric anaphylaxis. Traditional reliance on serum tryptase is being supplemented by novel biomarkers such as MCPT-1 and cytokine profiles, which may allow earlier detection and severity prediction. Component-resolved diagnostics further refine allergen identification and risk assessment, enabling individualized avoidance and prevention strategies. In acute emergency care, rapid diagnostic testing for infectious mimics (e.g., sepsis) supports stewardship while ensuring timely differentiation and intervention. This dual approach addresses both the immediate needs of the patient and the wider healthcare system burden [30].

Pharmacokinetics, pharmacodynamics, and precision dosing

Children differ significantly from adults in drug absorption, distribution, metabolism, and clearance, leading to variability in epinephrine exposure across age groups. These developmental differences highlight the limitations of uniform dosing and underscore the importance of model-informed precision dosing. Population pharmacokinetic and physiologically based modeling now guide individualized algorithms, while alternative delivery routes such as sublingual and intranasal formulations offer practical options that improve acceptability and adherence in pediatric emergencies.

Antimicrobial stewardship and drug-induced reactions

Drug-induced anaphylaxis, particularly from β-lactam antibiotics, remains a major contributor to pediatric emergency presentations. In the context of rising antimicrobial resistance, balancing immediate treatment of suspected infections with the risk of hypersensitivity is increasingly complex. Stewardship programs stress accurate allergy histories, avoidance of unnecessary broad-spectrum alternatives, and integration of rapid microbiology diagnostics to guide therapy. Therefore, tailored emergency protocols are essential not only for patient safety but also for preserving antimicrobial effectiveness.

Future directions

The trajectory of pediatric anaphylaxis care is shifting toward individualized, precision-based approaches. Translating mechanistic discoveries, such as mitochondrial regulation, immunomodulatory interventions, and biomarker-guided diagnostics, into practical bedside tools represents the next frontier. Integration of artificial intelligence-driven predictive models, real-world validation of alternative epinephrine delivery systems, and novel immunotherapies could transform both acute management and long-term prevention strategies. Interdisciplinary collaboration across emergency medicine, immunology, pharmacology, and microbiology will be critical to advancing these innovations.

## Conclusions

Pediatric anaphylaxis requires rapid recognition, timely intervention, and an individualized approach to management. Understanding its underlying mechanisms and the unique challenges of pediatric pharmacology supports more precise care. Ongoing innovations in diagnostics, delivery methods, and multidisciplinary strategies hold promise for safer and more effective treatment. The future of pediatric anaphylaxis management lies in translating these insights into practical, patient-centered tools that can be readily applied in emergency settings.

## References

[1] Piotin A, Oulehri W, Charles AL, Tacquard C, Collange O, Mertes PM, Geny B (2024). Oxidative stress and mitochondria are involved in anaphylaxis and mast cell degranulation: a systematic review. Antioxidants (Basel).

[2] Andrea D, López O (2023). Pediatric anaphylaxis: narrative review. Glob J Res Anal.

[3] Morriello F, Chapman M (2023). Epinephrine in anaphylaxis. CMAJ.

[4] Carlisle A, Lieberman J (2021). Clinical management of infant anaphylaxis. J Asthma Allergy.

[5] Klein O, Sagi-Eisenberg R (2019). Anaphylactic degranulation of mast cells: focus on compound exocytosis. J Immunol Res.

[6] Yang X, Lee D, Kim HW, Park BH, Lim C, Bae EJ (2024). Cannabidiol inhibits IgE-mediated mast cell degranulation and anaphylaxis in mice. Mol Nutr Food Res.

[7] Song J, Zheng J, Li Z (2024). Sulfur dioxide inhibits mast cell degranulation by sulphenylation of galectin-9 at cysteine 74. Front Immunol.

[8] Nagata K, Ando D, Ashikari T (2024). Butyrate, valerate, and niacin ameliorate anaphylaxis by suppressing IgE-dependent mast cell activation: roles of GPR109A, PGE2, and epigenetic regulation. J Immunol.

[9] Guilarte M, Sala-Cunill A, Luengo O, Labrador-Horrillo M, Cardona V (2017). The mast cell, contact, and coagulation system connection in anaphylaxis. Front Immunol.

[10] Goldman RD (2013). Acute treatment of anaphylaxis in children. Can Fam Physician.

[11] Kim H, Alizadehfar R, Alqurashi W (2023). Epinephrine autoinjectors: individualizing device and dosage to optimize anaphylaxis management in the community setting. Allergy Asthma Proc.

[12] Inoue N, Yamamoto A (2013). Clinical evaluation of pediatric anaphylaxis and the necessity for multiple doses of epinephrine. Asia Pac Allergy.

[13] Rachid O, Rawas-Qalaji M, Simons KJ (2018). Epinephrine in anaphylaxis: preclinical study of pharmacokinetics after sublingual administration of taste-masked tablets for potential pediatric use. Pharmaceutics.

[14] Li H, Fleischer D, Lockey R, Kaliner M, Lowenthal R, Tanimoto S (2023). A single-period, single-dose study of the pharmacokinetics of epinephrine after administration of intranasal ARS-1 to pediatric subjects with a history of systemic allergic reaction. J Allergy Clin Immunol.

[15] Short HB, Walters B, Fabi M, Ravida N, Boehmer S, Reyes L (2024). Evaluating practice patterns of observation periods following epinephrine administration for anaphylaxis among pediatric patients. Cureus.

[16] Xing Y, Zhang H, Sun S (2018). Clinical features and treatment of pediatric patients with drug-induced anaphylaxis: a study based on pharmacovigilance data. Eur J Pediatr.

[17] Meesters K, Buonsenso D (2024). Antimicrobial stewardship in pediatric emergency medicine: a narrative exploration of antibiotic overprescribing, stewardship interventions, and performance metrics. Children (Basel).

[18] Brockow K, Plata-Nazar K, Lange M, Nedoszytko B, Niedoszytko M, Valent P (2021). Mediator-related symptoms and anaphylaxis in children with mastocytosis. Int J Mol Sci.

[19] Kim AR, Jeon SG, Kim HR (2024). Preventive and therapeutic effects of Lactiplantibacillus plantarum HD02 and MD159 through mast cell degranulation inhibition in mouse models of atopic dermatitis. Nutrients.

[20] Tarczoń I, Cichocka-Jarosz E, Knapp A, Kwinta P (2022). The 2020 update on anaphylaxis in paediatric population. Postepy Dermatol Alergol.

[21] Montañez MI, Mayorga C, Bogas G (2017). Epidemiology, mechanisms, and diagnosis of drug-induced anaphylaxis. Front Immunol.

[22] Marsot A (2018). Pharmacokinetic variability in pediatrics and intensive care: toward a personalized dosing approach. J Pharm Pharm Sci.

[23] Galileya LT, Wasmann RE, Chabala C (2023). Evaluating pediatric tuberculosis dosing guidelines: a model-based individual data pooled analysis. PLoS Med.

[24] Freriksen JJ, van der Heijden JE, de Hoop-Sommen MA, Greupink R, de Wildt SN (2023). Physiologically based pharmacokinetic (PBPK) model-informed dosing guidelines for pediatric clinical care: a pragmatic approach for a special population. Paediatr Drugs.

[25] Wang K, Jiang K, Wei X, Li Y, Wang T, Song Y (2021). Physiologically based pharmacokinetic models are effective support for pediatric drug development. AAPS PharmSciTech.

[26] Gijsen M, Vlasselaers D, Spriet I, Allegaert K (2021). Pharmacokinetics of antibiotics in pediatric intensive care: fostering variability to attain precision medicine. Antibiotics (Basel).

[27] Snider CS, Hasara SL, Wilson KM, Glueck JA, Barbera AR (2023). Impact of outpatient antihypertensive medication use on epinephrine resistance in anaphylaxis. Cureus.

[28] Schaedeli Stark F, Chavanne C, Derks M (2024). A population pharmacokinetics model of balovaptan to support dose selection in adult and pediatric populations. J Pharmacokinet Pharmacodyn.

[29] Patel N, Isaacs E, Duca B, Nagaratnam N, Donovan J, Fontanella S, Turner PJ (2023). Optimal dose of adrenaline auto-injector for children and young people at risk of anaphylaxis: a phase IV randomized controlled crossover study. Allergy.

[30] Bhumireddy SK, Gudla SS, Vadaga AK, Nandula MS (2025). Vancomycin-induced DRESS syndrome: a systematic review of case reports. Hosp Pharm.

